# Options for tracking GFP-Labeled transplanted myoblasts using *in vivo* fluorescence imaging: implications for tracking stem cell fate

**DOI:** 10.1186/1472-6750-14-55

**Published:** 2014-06-12

**Authors:** Zhong Yang, Yaming Wang, Yanan Li, Qiang Liu, Qing Zeng, Xiaoyin Xu

**Affiliations:** 1 Department of Clinical Hematology, College of Laboratory Medicine, Southwest Hospital, Third Military Medical University, No. 29, GaoTanYan Street, ChongQing 400038, PR China; 2 Department of Anesthesia, Brigham & Women’s Hospital, Boston, MA 02115, USA; 3 Department of Radiology, Functional and Molecular Imaging Center, Brigham & Women’s Hospital, 75 Francis Street SR 153, Boston, MA 02115, USA

**Keywords:** GFP-Labeled Transplanted Myoblasts, Fluorescence Imaging, Stem Cell

## Abstract

**Background:**

Green fluorescent protein (GFP) is a useful biomarker, widely used in biomedical research to track stem cells after transplantation and/or to assess therapeutic transgene expression. However, both GFP and therapeutic gene products themselves may be immunogenic to the recipient. The main aim of this study was to use animal models to evaluate potential impact of GFP on the cell engraftment and to optimize tracking strategies prior to transplantation.

**Results:**

By using a fluorescent imaging (FLI) system, we investigated the dynamic cell behavior of GFP-transduced myoblasts in tibialis anterior (TA) muscles of immunocompetent *mdx* mice and immuno-compromised nude mice over a period of three months. The results suggested an apparent underlying host immunorejection in the *mdx* mice. Dystrophin immunostaining showed that the engraftment of wild type myoblasts was much more effective than that of the GFP-labeled counterparts in the *mdx* mice, further confirming an antigen role of GFP in this process. We tracked the GFP-transduced myoblasts in C57BL/6 mice and found GFP to be minimally immunogenic in these animals, as indicated by the GFP signal maintaining a much stronger level than that found in *mdx* and BALB/c mice at parallel time points. We also compared the *in vivo* cell behavior differences between myoblasts from virally GFP-transduced and GFP transgenic mice. The latter displayed much better engraftment, as determined both biomaging and histological observations.

**Conclusions:**

Our results not only demonstrated the immunogenicity of GFP in immunocompetent mice, but determined the optimized conditions for GFP-based *in vivo* stem cells tracking, that can potentially be extrapolated to human biomedical research.

## Background

Cell-based therapy in general and transplantation of stem cells in particular, is considered one of the most promising strategies for the treatment of several refractory diseases such as muscular dystrophies, neurodegenerative disorders or cardiac stroke. However, to date, besides the hematopoietic stem cell transplantation, cellular therapies are still far away from being routinely used for clinical purposes [1-3], and further research is needed to clearly define mechanisms of their pharmaceutical properties and fully understand their potential biological side effects. Therefore, there is a great need for *in vivo* imaging methods to dynamically track the location, survival and engraftment of transplanted cells [1,4-7].

GFP is a useful biomarker, widely used in different kinds of tissues to specifically track transplanted cells’ fate and/or validate the expression of therapeutic transgenes. In the past few years, advances in the field of optical molecular imaging expedited the use of GFP in broader areas of biomedical research [8-11]. Unfortunately, an unavoidable problem of this technique is the immunogenicity of GFP itself that has been described by several reports [12-15]. It is essential to be able to track and assess the engraftment of transplanted stem cells, as their properties and differentiation can be easily affected by multiple factors [1,8,16,17]. Even in a case of fully histocompatible or gene-modified autologous transplantation, many of the therapeutic gene-encoded proteins are immunogenic to the host, requiring administration of immunosuppressants [18-20]. As suggested by the previous studies in the field of tumor cell immunological rejection, the ideal transplantation host should be immunocompetent, with minimal immune response to the GFP reporter so as to obtain a better fluorescent signal [21,22]. The main objective of our study was to evaluate the immunogenic effect of GFP in several animal models, and to determine optimal conditions for GFP-based *in vivo* stem cells tracking.

Molecular imaging techniques offer a platform that can be used to monitor labeled transplanted cells in living animal not only non-invasively but also continuously and quantitatively. One of the clear advantages in using GFP as a biomarker for optical molecular imaging is that GFP-labeled cells can be directly examined under a fluorescent microscope after tissue harvest. In general, there are two main sources of the GFP-labeled cells: primary cultured cells that are retrovirally GFP-transduced ex vivo, and cells directly isolated from GFP-transgenic animals. So far, both of these sources of GFP-labeled cells are widely used for *in vivo* cell tracking and optical imaging [23-25]. However, for the same type of tissue stem cells, it remains unclear whether some observed discrepancies are a result of the different origin of GFP-labeled cells used, and whether one source should be preferentially chosen in the tracking experiments.

Myoblast transplantation is a cell-based therapy that aims to restore the normal structure of pathogenic muscle and to provide a source of stem cells for muscle repair, and is regarded as a unique way to deliver genes for therapeutic purposes [26,27]. In the present study, we used the fluorescent imaging in combination with histological observation to show that GFP plays a critical role in the immunorejection response to transplanted GFP-labeled myoblasts. Several animal models were used in this study, including the *mdx* mouse model for the Duchenne’s muscular dystrophy, that carries the mutation within the dystrophin gene [28], as well as the immunodeficient nude, wild-type BALB/c and C57BL/6 mouse model. We found that the C57BL/6 mouse was a better host candidate than other strains for GFP-labeled myoblasts transplantation because of its minimal immunogenic response to GFP. We demonstrated that GFP-transduced myoblasts possess myogenic differentiation potential and re-enter the satellite niche after transplantation. We also showed that the myoblasts from the GFP transgenic mouse display much more effective engraftment capability compared to *ex vivo* GFP-transduced ones. We believe that determining optimal conditions for GFP-based *in vivo* stem cells tracking may potentially contribute to further development of effective cell-based therapies in human subjects.

## Methods

### Animals

Imaging experiments used nude, mdx, C57BL/6, and BALB/c WT mice that were obtained from Charles River Laboratories. C57BL/Ka-β-actin-EGFP mice were a gift from A Wagers. The GFP transgenic-*mdx* hybrid strain was obtained by crossing the male *mdx* mice with female C57BL/Ka-β-actin-EGFP mice. Genotyping of dystrophin and GFP were performed using PCR (polymerase chain reaction). Adult (6–8 weeks) male mice were used in the study. All animal experimental manipulations were conducted in accordance with a protocol approved by the Institutional Animal Care and Use Committee of Harvard Medical School.

### Isolation of primary myoblasts and stable transduction of eGFP gene

The primary myoblasts were isolated from the leg muscles of adult C57BL/10 (an inbred substrain of C57BL/6) or C57BL/Ka-β-actin-EGFP mice using the protocol described by Rando and Blau [29]. After verifying their myogenic identity using immunocytochemistry with an anti-desmin antibody (1:200 dilution, Abcam Biochemicals, Cambridge, MA, USA), myoblasts from C57BL/10 were infected with eGFP-expressing lentiviral vectors. The eGFP-expressing myoblasts were cultured in Ham's F10 medium (Life Technologies, Carlsbad, CA, USA) supplemented with 20% of fetal calf serum (Life Technologies, Carlsbad, CA, USA) and 5 ng/ml basic fibroblast growth factor (Sigma, St Louis, MO, USA). Before cell transplantation, the eGFP expression in the myoblasts was verified using a fluorescence microscope. Cell were trypsinized, and after three washes with phosphate-buffered saline and the determination of cell number, the final cell pellet was suspended in Hanks solution to a final density of 2 × 10^7^–4 × 10^7^ cells/ml.

### Myoblasts transplantation

Six to eight week-old male mice were anesthetized by a cocktail of ketamine and xylazine in saline (100 mg/15 mg in 5 ml saline) at a dosage of 100 μl/20 g of body weight. The mouse was placed in a prone position. Non-nude mice leg hair was removed using Nair, a commercially available hair remover (Church & Dwight, Princeton, NJ, USA), and then the leg was wiped with cotton swap dipped in clean water. Using a Hamilton syringe, a total of 5 × 10^5^ eGFP-labeled myoblasts in 20 μl of Hanks were evenly injected at three positions along the axis of tibialis anterior (TA) muscle. After cell injection, the needle was slowly withdrawn in 3 minutes.

### Fluorescence imaging station

We used a planar fluorescence imaging station, NightOwl LB981 (Berthold Technologies USA, LLC., Oak Ridge, TN), to acquire the fluorescent imaging (FLI) data and its software toolbox, WinLight 3.2, to process the images. The imaging station features a charge-coupled device (CCD) camera of high sensitivity that can be moved in a vertical direction for optimal focus. The imaging chamber is light-tight to reduce background noise. For the imaging of eGFP, an illumination light source passes through a band-pass filter with a central wavelength of 475 nm to excite the protein, and a band-pass detection filter with a central wavelength of 525 nm selectively captures the emission light of the excited eGFP. The excitation and detection filters have a full-width half magnitude of 40 and 10 nm, respectively. Light photographs of the subjects can be taken by switching off the detection filter.

### Imaging procedure and quantification of imaging data

Mice were anesthetized using the protocol described above. Non-nude mice leg hair was removed using Nair as described above. Each mouse was placed in a prone position on a piece of nonfluorescent black paper (Strathmore Series 400). To ensure that TA muscles were imaged in the same position, both legs were positioned 180° apart from each other and 90° relative to the body axis. Both feet were taped to the paper with their dorsal sides up. The CCD camera was set for a field of view of 6.5 cm^2^ with an estimated specimen height of 0.9 cm. A photographic picture was taken at an exposure time of 10 seconds followed by a fluorescence image at an exposure time of 1,250 ms.

We quantified the measurements with both the normalized photon counts and the area of eGFP signals. The region of interest (ROI) containing eGFP^+^ signal in the fluorescence images was outlined manually, and then the intensity (photon counts/second/mm^2^) of the ROI as well as the area of the ROI was recorded. To normalize the fluorescent signal from autofluorescence, we also measured the intensity of an adjacent eGFP^−^ region. We then subtracted the photon counts/second/mm^2^ of ROI by the photon counts/second/mm^2^ of the eGFP^−^area and calculated the total photon counts generated by eGFP^+^ cells by timing the normalized intensity within the area of the eGFP^+^ region.

### Muscle harvest, histology and immunohistochemistry staining

Mice were anesthetized with xylazine and ketamine as described above and had their left ventricles perfused with 4% (w/v) paraformaldehyde (PFA, Sigma, St Louis, MO, USA) in phosphate-buffered saline (PBS) (pH 7.4). The TA muscles were then carefully removed. Tissues were post-fixed in 4% PFA over 12 hours, submersed in 30% sucrose overnight, frozen in OCT embedding compound, and sectioned coronally (10 microns) with a cryostat. Cryo-sections were thaw mounted onto gelatin-coated slides and stored at -20°C.

Frozen sections, after being rinsed three times in PBS, were permeabilized and blocked with PBS containing 0.1% (v/v) Triton X-100 and 10% (v/v) goat serum in PBS for 1 hr at room temperature (RT) and then incubated with either rabbit-anti-dystrophin antibody (1:600, Abcam Biochemicals, Cambridge, MA, USA) or mouse-anti-Pax7 antibody (1:50, Hybridoma Bank, Iowa City, IA, USA). The sections were then incubated with the goat-anti-rabbit CyTm5-conjugated or goat-anti-mouse Cy3-conjugated secondary antibody (Jackson Lab) for 1 hr at RT. Slides were counterstained with DAPI dye (1:800, Sigma, St Louis, MO, USA), rinsed and coverslipped with fluorescence mounting medium (Dako, Glostrup Denmark). Stained sections were examined under the Olympus fluorescence microscope (Olympus, Center Valley, PA, USA), and digital images of sections were acquired with a CCD camera.

### Statistical analysis

Quantitative data were presented as a means ± standard deviation (SD). A normal distribution of the samples was confirmed and a student’s t-test (assuming equal variances) was performed to determine the statistical significance between two experimental groups. A P value less than 0.05 was considered to be statistically significant.

## Results

### Transplanted GFP-labeled myoblasts display different engraftment pattern in mice with different immune backgrounds

**Figure 1:**
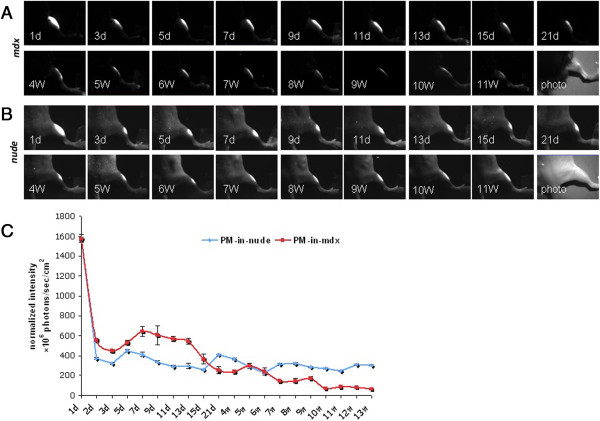
**Dynamic engraftment of transplanted GFP-labeled myoblasts in *tibialis anterior *(TA) muscle of *mdx *and nude mice shown by *in vivo *fluorescent imaging.** The last picture in A and B is the bright-field photo. **(A)** A representative of dynamic TA muscle imaging in *mdx* mouse. **(B)** A representative of dynamic TA muscle imaging in nude mouse. **(C)** Quantitative data of the GFP fluorescence intensity in *mdx* and nude mice. PM, GFP-labeled primary myoblast. n = 6.

In the FLI study, we initially investigated the dynamic engraftment of GFP-labeled myoblasts in TA muscles of *mdx* and immunodeficient nude mice groups for 11 weeks after transplantation. The results revealed that during the first 6 weeks fluorescent signals showed similar changes in the two groups. The TA muscle exhibited its highest fluorescent intensity just after transplantation. Following transplantation, the fluorescent intensity decreased by more than 80% compared to the highest value at 72 h. Then it increased rapidly in the following week, and gradually declined thereafter. After 6 weeks, the intensity remained basically stable in the immunodeficient group, at around 23% of its original value. In contrast, in immunocompetent *mdx* mice, the fluorescent intensity further weakened to less than 6% of the original value at week 11, suggesting that the immunological rejection response of the host probably played a role in the loss of transplanted myoblasts (Figure 1A-C). This response may be triggered either by GFP or by dystrophin (the latter of which would be regarded as a transgene to the mdx mouse).

### Reverse transplantation study provides explicit evidence of the immunogenic role of GFP

**Figure 2:**
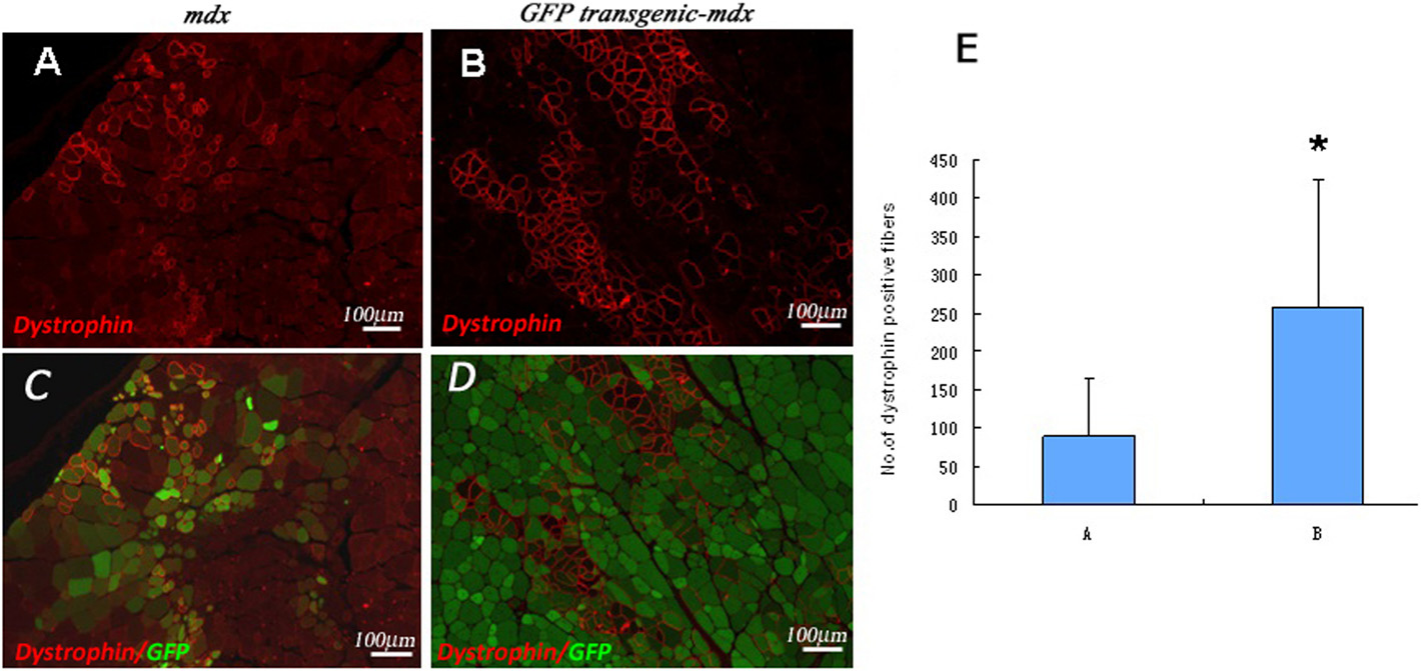
**Dystrophin immunostaining (red) showing the engraftment of transplanted myoblasts in dystrophin-deficient mice.** A TA muscle was injected with 5 × 10^5^ cells and harvested 2 months after transplantation. **(A)** TA muscle of *mdx* mouse injected with GFP-labeled myoblasts. **(B)** TA muscle of GFP transgenic-*mdx* hybrid mouse injected with non-labeled myoblasts. **(C)** Merged image of A with GFP. **(D)** Merged image of B with GFP. Scale bar = 20 μm. **(E)** Levels of dystrophin-positive fibers in TA muscle of *mdx* mouse injected with GFP-labeled myoblasts (bar A) and of GFP transgenic-*mdx* hybrid mouse injected with non-labeled myoblasts (bar B). Values are mean ± standard errors of 5 samples. (*P < 0.01; Student’s t-test).

To assess the effect of allogenic protein GFP on the survival of transplanted myoblasts, we used *mdx*-GFP hybrid mice. This strain was produced by crossing male *mdx* with female C57BL/Ka-β-actin-EGFP mice and genotyped before experiment. In this animal model, we transplanted wild type myoblasts passaged *in vitro* for the same generations as the GFP-modified ones into TA muscles. It excluded the immuno-rejection effect of GFP and was termed reverse transplantation. After one and two months, muscle samples were harvested, and the presence of dystrophin was measured by immunohistochemistry. The dystrophin positive fibers usually displayed a weak green color due to fusion with the GFP positive host fibers in the *mdx*-GFP hybrid mice. In the *mdx* mice, the dystrophin positive fibers generally overlapped with the GFP-labeled cells (Figure 2A, B), and a relatively denser infiltration of inflammatory cells could be observed around the dystrophin positive fibers (data not shown). Data showed that at two months after transplantation, *mdx*-GFP hybrid mice exhibited 2.9 fold higher levels of dystrophin positive fibers than *mdx* mice (257.6 ± 166.8 *versus* 88.7 ± 75.2) (Figure 2C).

### Transplanted GFP-labeled myoblasts engraft much better in the TA muscle of C57BL/6 mice than in other mice strains

**Figure 3:**
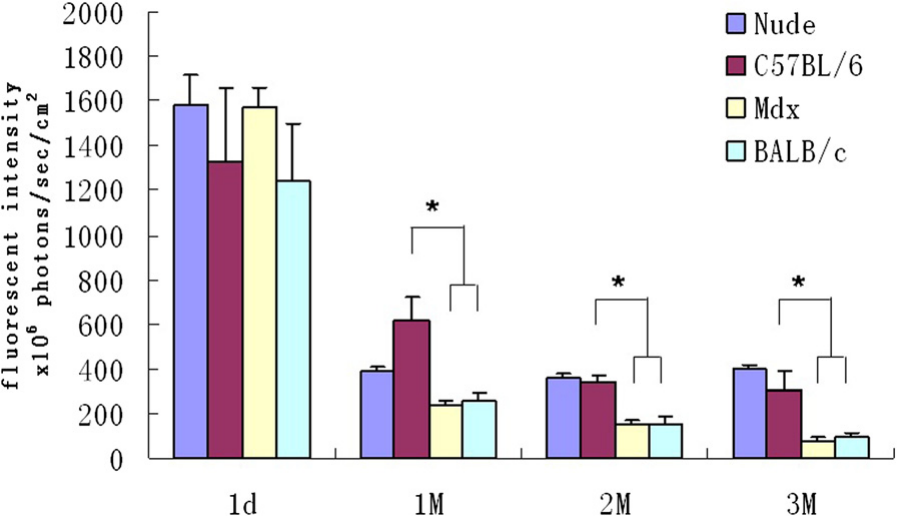
**Fluorescence intensities of TA muscles in of nude, C57BL/6, BALB/c and *mdx *mice.** *P < 0.01, Values are mean ± standard errors of 5 samples.

To explore the more suitable experimental host for the GFP-labeled cells, we further investigated the survival rate and engraftment efficiency of GFP-labeled myoblasts in C57BL/6 and BALB/c WT mice. Previous tumor cell inoculation study indicated that the immunogenicity of GFP is very low in C57BL/6 mice [21]. When transplanted with the same number of myoblasts (5 × 10^5^) into the TA muscles, the fluorescence intensity of the TA muscles in C57BL/6 mice was significantly higher than that in the BALB/c and *mdx* mice, and slightly lower than that in the nude mice at one, two and three months after transplantation. Compared with the original value, the fluorescence intensity in all groups significantly decreased one month post-transplantation. However, C57BL/6 mouse model maintained much higher and stable level of fluorescence intensity throughout the experiment, suggesting that the GFP-labeled myoblasts were minimally rejected in these animals. C57BL/6 mouse, therefore has been considered a favorable animal model for research on GFP-labeled cells transplantation (Figure 3).

### Myoblasts from the GFP-transgenic mouse exhibit much stronger engraftment capability than that from virally GFP-transduced *ex vivo*

**Figure 4:**
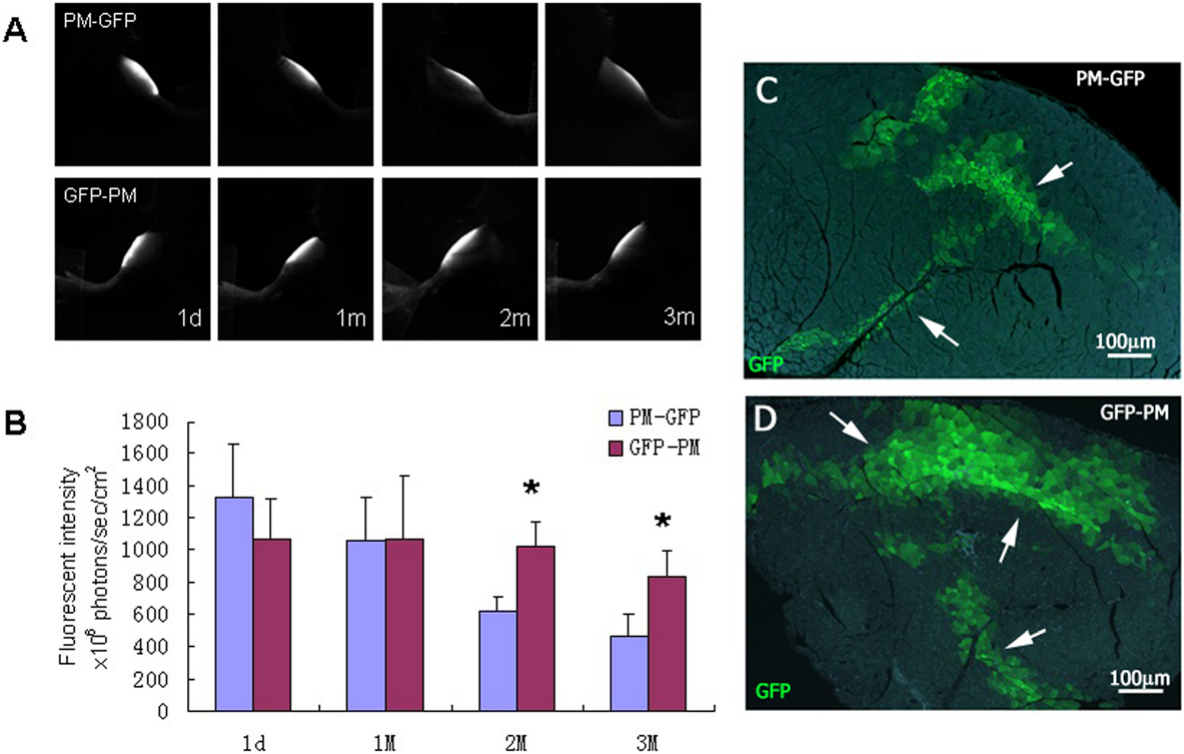
**The effect of different origin of GFP-labeled myoblasts on the engraftment in C57BL/6 mice. (A)** Representative dynamic TA muscle imaging of GFP-labeled primary myoblasts from transfected *in vitro* (PM-GFP) and from GFP transgenic mouse (GFP-PM). **(B)** Fluorescence intensity levels of the PM-GFP and GFP-PM. Data represent means and standard errors of 6 samples; *P < 0.01. **(C,D)** Histological examination of the TA muscle 1 month after transplantation with PM-GFP (upper panel) and GFP-PM (lower panel). Scale bar = 100 μm.

Next, we evaluated the impact of the GFP-labeled myoblasts derived from different sources on their survival and integration after transplantation in the C57BL/6 mice. Using FLI, we examined the cell behavior of transplanted myoblasts that were obtained either from the GFP transgenic mice or, alternatively, from *in vitro* transfection with a lentiviral vector containing GFP genes. Fluorescence intensities of the GFP-labeled myoblasts were measured in multi-well culture plates and the intensity of the equal number of cells was standardized for *in vivo* fluorescence imaging. Cell viability of myoblasts from GFP transgenic mice was significantly higher than cells obtained through the GFP-lentiviral transfection. After transplantation, GFP-labeled myoblasts from transgenic mice presented a stronger fluorescent signal over a wider area, especially at month one and two (Figure 4A, B).These data were further confirmed by analyzing the fluorescence signal of the muscle tissue sections. The green muscle fibers derived from myoblasts of GFP transgenic mice displayed a wider distribution and were more uniform in morphology one month post-transplantation, indicating that myoblasts from GFP-transgenic mice display higher differentiation potential. In contrast, many small and medium-sized cells with green fluorescence could still be observed in the GFP-lentivirus transfection group during the first two months (Figure 4C).

### GFP-transduced myoblasts not only display myogenic differentiation but produce donor-derived satellite cells after transplantation

**Figure 5:**
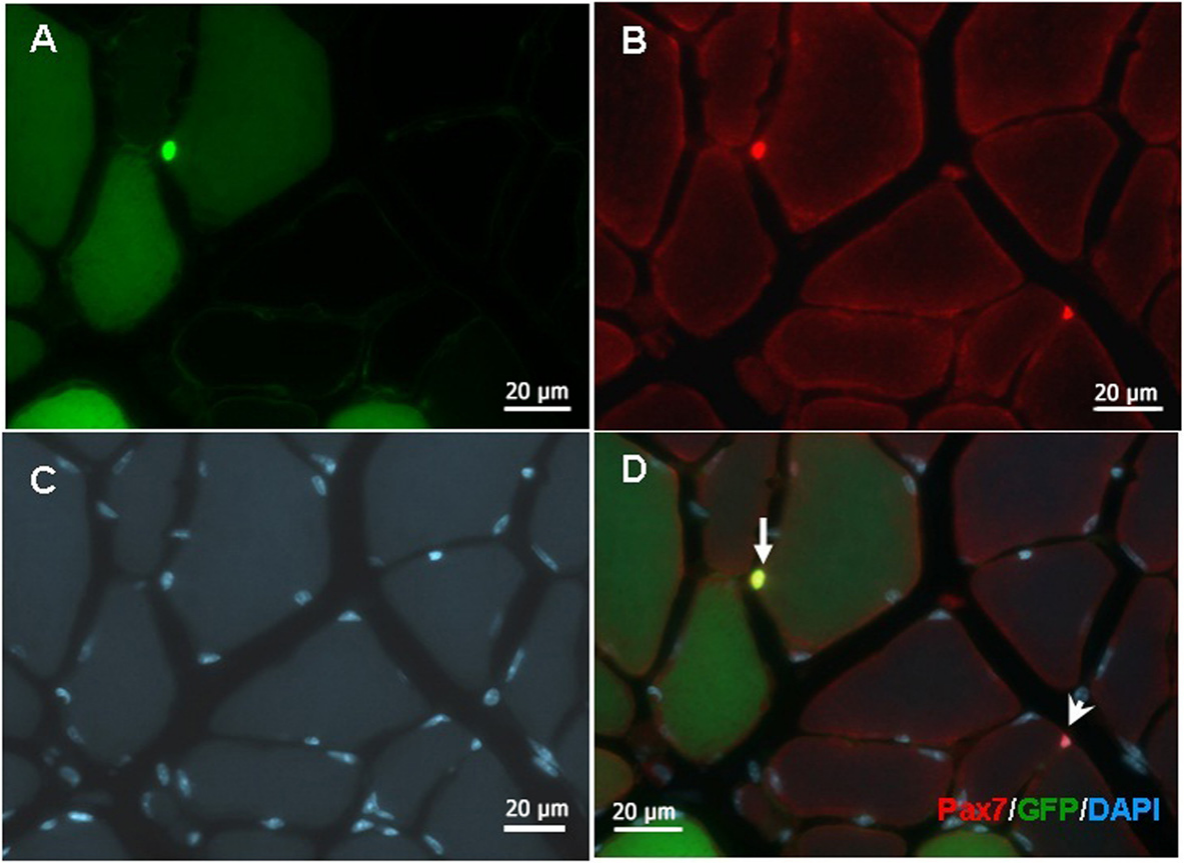
**Histological evidence of transplanted myoblast-derived satellite cells.** The TA muscle was harvested 1 month after transplantation and cryo-sectioned in 10 μm sections. **(A)** Green channel image shows the eGFP-labeled transplanted cells. **(B)** Anti-Pax7 immunostaining (red) shows 2 positive cells. **(C)** DAPI staining show the nuclei of all cells in this area. **(D)** Merged image of A, B and C. Arrow indicates a transplanted myoblast-derived satellite cell, arrowhead indicates a satellite of the host. Scale bar = 20 μm.

To evaluate the myogenic differentiation potential of GFP-transduced myoblasts, and especially their capability to generate donor-derived precursor cells of transplanted myoblasts, we examined the engraftment of GFP-labeled cells in TA muscles of immunodeficient nude and C57BL/6 mice by immunofluorescent histochemistry two months after transplantation. Results showed that when transplanted with 5 × 10^5^ myoblasts, hundreds of skeletal muscle fibers marked with GFP could be observed in TA muscles. The engrafted fibers were similar in size to the recipient and displayed green color in various shades based on how they fused with the host fibers. Very few centrally-located nuclei myofibers were observed at that time point. We detected small GFP-positive cells, redistributed in the microenvironment of satellite cells between the basement membrane and myofiber. These cells expressed satellite cells-specific marker, Pax7, indicating that the grafted GFP-labeled myoblasts could not only differentiate to form myofibers, but also reenter in in situ niche to constitute a stored stem cell pool (Figure 5).

## Discussion

Longitudinal follow-up of the fate of transplanted cells or transgene expression is critical for cell transplantation and gene therapy research. In recent years, *in vivo* imaging and tracking based on the GFP reporter gene has gradually become one of the most favorable strategies for such studies [1,11,30,31]. Several types of mutant GFP with good stability and enhanced fluorescent brightness have already been commercialized. The enhanced GFP (eGFP) gene in the present study is under the control of the promoter of the conservative actin gene, its fluorescent intensity is kept stable during the culture process and after *in vivo* transplantation. In a previous study, we monitored the dynamic process of skeletal muscle regeneration by transplanting EGFP-labeled myoblasts in severe combined immune deficiency (SCID) mice, and verified high sensitivity and reliability of this experimental approach [32]. However, determining the potential impact of GFP on the cell engraftment and optimizing the strategy for GFP-based in vivo tracking in disease or immunocompetent animal models, still remains a challenge.

As an exogenous protein, GFP marker was documented to be immunogenic [12,14,15]. In the present study, we observed the dynamic processes of transplanted GFP-labeled myoblasts in mice with different immunological backgrounds, as well as reverse transplantation of normal non-labeled myoblasts into *mdx*-GFP hybrid mice. Our results not only highlighted the immunogenic role of GFP, but also offered a way to assess the effect of transgene (dystrophin) on the survival of transplanted myoblasts.

In optical molecular imaging, consistent expression of the reporter gene is an important prerequisite for the tracking of transplanted living cells. However, the GFP itself was always immunorejected in the immunocompetent host as an exogenous protein. Skelton et al. first reported that GFP was less immunogenic to C57BL/6 mice in which the GFP-transduced EL-4 lymphoma cells could form subcutaneous tumors. However, they hypothesized that this phenomena might be an exception to the rule because the GFP-labeled tumors were rarely noted in other animals or relative species [21,22]. Our study showed that the fluorescent intensity of GFP-labeled myoblasts after transplantation in C57BL/6 mice was higher than that found in BALB/c and mdx mice, similar to the levels detected in nude mice, confirming that the GFP-transduced myoblasts were only minimally rejected in C57BL/6 mice. To our knowledge, this is first report suggesting that the GFP-transduced stem cells engraft better in C57BL/6 mice than in other species. Furthermore, low rejection rate of GFP-transduced myoblasts in this animal model means that there is no need for additional immunosuppressive agents that may potentially disturb the tissue homeostasis. We suggest therefore, that C57BL/6 mice are a more suitable host animal model for studies that require GFP-labeled stem cells tracking.

In addition to the early massive cell death, host versus graft reaction (HVGR) is a critical determinant of the success of cell transplantation. On condition that a recipient’s genetic background and major histocompatibility are coincident with the donor’s, the immunological response of hosts depends mainly on the structure and the dose of exogenous protein, the route of entry, the type of cells expressing the transgene as well as the viral vector system [16,33,34]. In the present study, we show that transplanted myoblasts from GFP-transgenic mice have better engraftment capacity comparing to *ex vivo* transfection. We used lentivirus vector to obtain *ex vivo* GFP-transduced myoblasts and obtained highly purified GFP-labeled cells using drug-resistance gene screening and flow cytometry sorting. GFP-labeled myoblasts were also isolated and cultured from transgenic mice in which the GFP gene was stably integrated into the genome at embryonic stage. This method allowed to avoid issues related to the immunogenicity and virulence of the viral vector in the virus-based transfection. It also didn’t have any related toxicity associated with viruses, and thus did not perturbed gene delivery. Consistent with this, studies from Ghazizadeh group indicated that re-transplanting in vitro GFP transfected keratocytes into the host significantly reduced the immune reaction compared with direct injection of the GFP gene, a phenomenon that may be attributed to the lack of the extra antigen brought by *in vivo* injection of the viral vector [35]. Recently, another study demonstrated that the vector-associated inflammation was reduced through *ex vivo* gene delivery in lung transplantation [36].

In summary, by combining the use of *in vivo* FLI, histology and immunohistochemistry, we not only demonstrated the immunogenicity of GFP reporter during cell transplantation, but more importantly, provided optimized conditions for GFP-based tracking using *in vivo* FLI. We also demonstrated that the immunocompetent C57BL/6 mouse is a better transplant recipient as it shows only minimal immunological rejection of GFP, and GFP transgenic mice appear to be a are better quality cell source than the *in vitro* transfected ones due to their ability to circumvent the immune response to the vector.

## Conclusions

GFP reporter has a distinct immunogenic effect in immunocompetent animal host during cell transplantation. Among several mouse models, C57BL/6 mouse is clearly a better transplant recipient with only minimal immunological rejection of GFP. It appears that GFP transgenic mice are able to evade the immune response to the vector, and therefore, may be considered a better quality cell source, while the use of *in vivo* FLI provides optimal conditions for GFP-based cell tracking and can potentially be extrapolated in human biomedical research.

## Competing interests

The authors declare that they have no competing interests.

## Authors’ contributions

ZY carried out animal procedures, FLI, and molecular studies, participated in the study design, statistical analysis and manuscript preparation; YW, YL, QZ: carried out molecular studies, performed statistical analysis, carried out animal procedures. XX: participated in study design, drafted the manuscript, and performed statistical analysis. All authors read and approved the final manuscript.
